# Renal nerve stimulation modulates renal blood flow in a frequency-dependent manner

**DOI:** 10.21203/rs.3.rs-7724105/v1

**Published:** 2025-10-14

**Authors:** Dzifa Kwaku, Dusty Van Helden, Joan Dao, John Osborn, Matthew D. Johnson

**Affiliations:** 1 Department of Biomedical Engineering, University of Minnesota; 2 Department of Surgery, University of Minnesota

**Keywords:** renal nerve, renal blood flow, peripheral nerve stimulation, kilohertz-frequency stimulation, blood pressure, hypertension

## Abstract

**Background:**

Chronic overactivity of the renal nerves is a key pathophysiological attribute of drug-resistant hypertension. Indeed, catheter-based renal denervation can lower blood pressure by severing the brain-kidney (efferent nerves) and kidney-brain (afferent nerves) link, but its irreversibility and potential nerve reconnection limits adaptability and longevity, highlighting the need for alternative treatments. Kilohertz-frequency electrical stimulation is an approach known to reversibly inhibit peripheral nerve activity and has potential to reversibly modulate renal blood flow.

**Methods:**

This study investigated how electrical stimulation of the renal nerves affects renal blood flow in a non-diseased anesthetized swine. Using unilateral hook electrodes around the renal artery complex, we performed parameter sweeps of stimulation frequency (20 −15000 Hz) and measured blood flow and blood pressure changes in the ipsilateral kidney.

**Results:**

Stimulation at low frequencies (≤100 Hz) resulted in a sustained reduction in renal blood flow. High stimulation frequencies (>100 Hz) often resulted in an immediate decrease in blood flow through the kidneys, but the responses exhibited adaptation with continued isochronal pulsatile stimulation. Notably, while kilohertz-frequency stimulation did not directly increase renal blood flow in this experiment, it did induce a carryover effect on renal nerve sensitivities to low-frequency stimulation, reducing the effect of subsequent low-frequency stimulation on renal blood flow (from −11.8% to −4.7%, median).

**Conclusions:**

Responses to renal nerve stimulation depend on stimulation frequency with effects that can persist or adapt as well as effects that can range from decreasing renal blood flow to decreasing the sensitivity of the renal nerve signaling pathway. These findings have important implications for future development of bioelectronic interfaces with the renal nerve for modulation of kidney function.

## Background

Hypertension is a major global public health concern, affecting approximately one-third of adults worldwide and significantly contributing to cardiovascular morbidity and mortality(1). Uncontrolled hypertension substantially elevates the risk for end-stage renal disease, ischemic heart events, heart failure, stroke, and mortality(2,3). While antihypertensive drugs are the primary therapy, a subset of hypertensive patients (10–20%) exhibit drug-resistant hypertension(4).

Among the key contributors to drug-resistant hypertension is the dysregulation of the autonomic nervous system and in particular renal sympathetic nerve overactivity(5,6). Renal nerve signaling, through afferent and efferent pathways, plays an important role in kidney function for the long-term regulation of arterial blood pressure forming a self-regulated renorenal reflex loop(7,5,8,6). Afferent feedback from the kidney provides state information to the brain, whereas efferent input to the kidney acts to modulate kidney function(6). Animal studies have shown that increased sympathetic nerve activity to the kidneys contributes to a rise in blood pressure through an increase in tubular reabsorption of urinary sodium and water resulting in sodium retention, reduction in renal blood flow (RBF) and glomerular filtration rate (GFR), and release of renin leading to the activation of renin angiotensin-aldosterone cascade, which further elevates systemic vascular resistance(9). As a result, interventions targeting the renal sympathetic nerves serve as potential therapeutic strategies for patients with drug-resistant hypertension.

Renal denervation (RDN) has emerged as a promising interventional approach that uses bilateral catheter-based radiofrequency ablation of the renal nerve to disrupt sympathetic signaling to the kidney and in turn lower blood pressure(10). While early pilot clinical trials showed a strong reduction in blood pressure, subsequent clinical studies have revealed inconsistent outcomes, with some patients experiencing limited or no sustained benefit(11). Such variability in clinical responses highlights the need for improved patient selection criteria, enhanced procedural technique, better understanding of renal nerve function, and better assurance that the ablation procedure was successful in targeting the renal nerves.

To address variability in RDN outcomes, recent studies have explored the prognostic and diagnostic utility of renal nerve stimulation (20 Hz) in humans and animal models(12–15). This method uses low-frequency electrical stimulation (LFS, <100 Hz, isochronal pulse trains) within the renal artery to determine which locations along the renal artery result in target engagement of the renal nerves and thus may be receptive to subsequent ablation. Despite these advances, there remains a limited understanding of the optimal stimulation parameters for consistent and predictive outcomes(16).

Another approach to selectively block nerve conduction in peripheral nerves involves the use of kilohertz-frequency stimulation (KHF)(17). KHF has been widely studied in pain management(18), regulating autonomic functions(19), and treating conditions such as obesity through vagus nerve stimulation(20,19,21), but has not been systematically investigated when applied to the renal nerves, representing a critical gap in knowledge.

The primary objective of this study was to determine how the frequency of renal nerve stimulation influences renal blood flow, focusing on the potential of KHF to provide a novel, non-destructive form of neuromodulation therapy. Based on previous preclinical and clinical studies, we hypothesized that low-frequency stimulation would reduce blood flow to the kidney, due to enhanced sympathetic activation, and KHF would initially reduce renal blood flow followed by a compensatory increase in renal blood flow.

## Methods

### Animal Model

Seven Yucatan miniature pigs (1 female, 6 males, 42.5 +/− 4.5 kg, 5– 8 months old) were used in this study. All animals were healthy and were maintained on a Teklad 7200 Vegetarian Pig/Sow Grower Diet (Inotiv, Madison, WI) before surgery. The Yucatan pig model was used in this study as it is considered a suitable model for evaluating renal sympathetic denervation approaches(22). All experiments were performed in compliance with the institutional care and use committee at the University of Minnesota and with the United States Public Health Service policy on the humane care and use of laboratory animals.

### Experimental Preparation

The animals were initially sedated with Telazol (2–8 mg/kg, IM) and Xylazine (0.4–1.6 mg/kg, IM). After allowing time for sedation, the ear vein was aseptically prepped for catheter placement, and an intravenous catheter was inserted and connected to a 0.9% normal saline drip. Anesthesia was induced using Propofol (1–2 mg/kg, IV) and followed by endotracheal intubation and maintenance on inhaled isoflurane (2–3%) for the duration of the procedure. With the animal in a supine position, the left kidney was exposed through a midline abdominal incision. Fat and connective tissue around the left renal artery was bluntly dissected to access the renal nerves, taking care not to damage nerves both near the kidney as well as at the junction of the renal artery and descending aorta.

Hook electrodes (made from insulated tungsten wire, 203 μm wire diameter, A-M Systems) were constructed by shaping the exposed wire around a 4 mm diameter arc. Two hook electrodes were surgically inserted into the tissue and around the renal artery-nerve complex, with the hook shape serving to anchor the electrode in place. Placement of the hook electrodes varied amongst the subjects but was primarily targeted to the renal nerves near the junction between the renal artery and the descending aorta. The spatial separation between the hook electrodes was 5 −10 mm.

An ultrasound transit-time flow probe (Model 6PSB1377, 6 mm diameter, Transonic Systems Inc., Ithaca, NY) was placed around the renal vein to measure renal blood flow exiting the kidney. Additionally, an arterial catheter was inserted into the femoral artery and connected to a DTX-Plus pressure transducer (Model 682018, Merit Medical Singapore Pte., Ltd) to monitor changes in blood pressure. Signals from both the flow probe and the blood pressure transducer were sampled at 1 kHz, digitized at 16-bit resolution, and recorded using a PowerLab 16/35 data acquisition system (ADInstruments, Colorado Springs, CO).

### Electrical Stimulation

**Figure 1.**
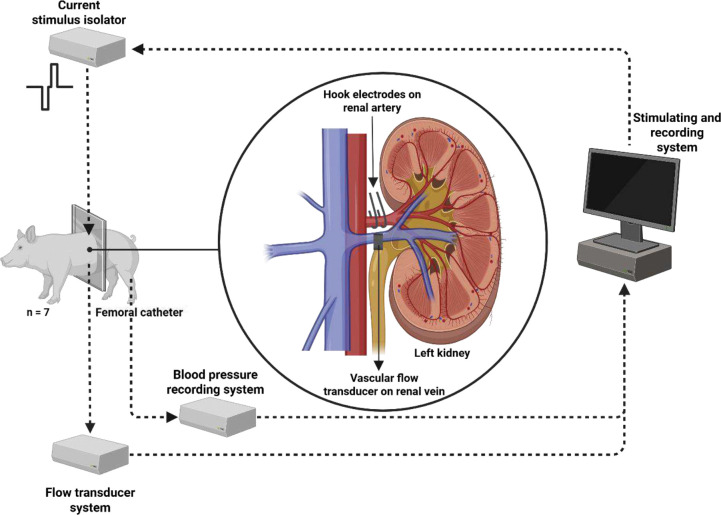
Experimental setup for renal blood flow recordings during renal nerve stimulation. Schematic representation of the *in vivo* setup in anesthetized pigs. Electrical stimulation was delivered via hook electrodes placed around the left renal nerve-artery complex. Renal blood flow was continuously measured using an ultrasound transit-time flow probe placed on the left renal vein. A femoral arterial catheter was inserted for systemic monitoring. The configuration preserved perivascular connective tissue to maintain native anatomical relationships.

The hook electrodes were connected to the output of a stimulus isolator (A-M Systems, Model 2200), which was controlled by a function generator (analog output, Intan RHS Stim/Recording System) to create the stimulus pulse trains shown in Figure 1. The study used randomization of nerve stimulation frequencies to mitigate sequential effects of stimulation settings. Additionally, the study included control trials involving periods of no stimulation to verify that the stimulus-induced effects were not related to random chance and to verify that the wash-out period was sufficient for RBF to return to pre-stimulation levels. Two electrode configurations were evaluated in each pig to determine the most effective stimulation. These included a (1) monopolar configuration, in which stimulation was delivered through the hook electrode and returned via a needle electrode inserted in the abdominal muscle wall, and a (2) bipolar configuration, in which the stimulation was delivered and returned through the adjacent hook electrode around the renal artery complex. Comparative testing revealed that the monopolar configuration showed the most pronounced changes in renal blood flow, likely due to a broader spread of current. After placing the hook electrodes, tuning of stimulation amplitude occurred with either 20 Hz or 50 Hz pulse trains delivered for 30 – 60 seconds. In four of the pigs, stimulation amplitudes ranging from 0.5 to 7 mA were randomly applied, and the highest amplitude setting was chosen given that it produced the largest decrease in blood flow. In the other three pigs, a stimulation amplitude of 2.5 mA was used.

### Experimental Protocol

The first experiment investigated a parameter sweep of stimulation frequencies (20, 50, 100, 200, and 1000 Hz) through the hook electrodes. Stimulation was applied for 30 – 120 seconds followed by a wash-out period of 60 – 120 seconds of no stimulation to allow the blood flow rate to return to baseline. A second experiment was performed to investigate the effects of kilohertz frequency stimulation. This involved first applying a low-frequency (20 or 50Hz) test stimulation pulse train for 30 – 60 seconds, followed by KHF (1000 Hz or 15000 Hz) for 1 – 5 minutes, and then another low-frequency re-test stimulation pulse train (20 or 50Hz) for 30 – 60 seconds. A control experiment was also performed with a low-frequency stimulation test and re-test conditions without the 1 – 5 minutes of KHF. For both protocols, the stimulus waveform was charge-balanced and biphasic. For frequencies below KHF, pulses were cathodic-first with 500 μs duration and 100 μs interphase gap. For all KHF stimulation, pulses had no interphase delay: 1000 Hz used 500 μs per phase, while 15000 Hz used 33.3 μs per phase.

### Hemodynamic analysis

All renal blood flow data were processed using MATLAB 2025a (MathWorks). Pre-processing involved applying a 4^th^-order Chebyshev Type II low-pass filter to minimize breathing artifacts in the blood flow signals (cutoff frequency: 0.15 Hz, stopband attenuation: 20 dB, poles inside unit circle at |p|≈0.999). Given fluctuations in blood flow between diastolic and systolic intervals, data were also averaged over 5 second windows in both baseline (pre-stimulation) and stimulation conditions.

Two key metrics were computed for the short- and longer- term dynamics of renal blood flow responses to renal nerve stimulation across pulse train frequencies:

1. **Initial response** assessed the immediate blood flow change ($△RBF_{\mathrm{initial}}$) by comparing the mean blood flow 2–7 seconds after stimulation onset to baseline (−10 to −5 seconds) before stimulation onset.
$$\Delta RBF_{\mathrm{initial}}=\left(\frac{{\bar{RBF}}_{2:7}-{\bar{RBF}}_{-10:-5}}{{\bar{RBF}}_{-10:-5}}\right)\times 100\%$$
2. **Extended response** assessed the temporal changes in blood flow ($△RBF_{\mathrm{extended}}$) by comparing the mean blood flow 45 – 50 seconds after stimulation onset to the mean blood flow 2–7 seconds after stimulation onset.
$$\Delta RBF_{\mathrm{extended}}=\left(\frac{{\bar{RBF}}_{45:50}-{\bar{RBF}}_{2:7}}{{\bar{RBF}}_{2:7}}\right)\times 100\%$$

The persistent effects of KHF on renal blood flow were also calculated as average responses of low-frequency stimulation responses before and after delivering 1–5 minutes of KHF normalized to baseline.


$$\Delta RBF_{\mathrm{persistent}}=\frac{{\bar{RBF}}_{\mathrm{pre/post}}}{{\bar{RBF}}_{\mathrm{baseline}}}=\left(\frac{{\bar{BF}}_{0:30}}{{\bar{BF}}_{-10:-5}}\right)\times 100\%$$

### Statistical analysis

We hypothesized that 20 Hz stimulation would decrease renal blood flow, whereas 15000 Hz stimulation would increase renal blood flow. This hypothesis was evaluated using a Mann-Whitney U test (p<0.05). For exploratory analyses, we utilized a Kruskal-Wallis test with Bonferroni post-hoc corrections to compare the initial and extended responses across multiple stimulation frequencies (20, 50, 100, 200, 1000, and 15000 Hz) to control (no stimulation). Additionally, a Wilcoxon signed-rank test was conducted to test the hypothesis that renal blood flow responses to low-frequency stimulation would be lower after kilohertz-frequency stimulation.

## Results

### Initial renal blood flow response depends on renal nerve stimulation frequency

**Figure 2.**
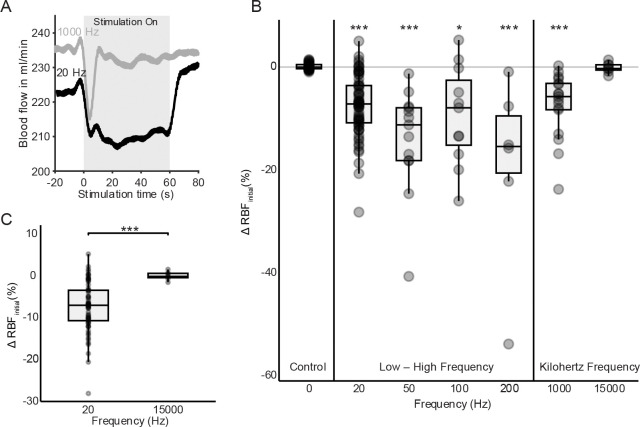
Frequency-dependent initial changes in renal blood flow (RBF) during renal nerve stimulation. **(A**) Representative RBF traces from a single subject showing blood flow responses during 20 Hz and 1000 Hz stimulation. Low-frequency stimulation (20 Hz) resulted in a sustained reduction in flow, whereas kilohertz-frequency stimulation (1000 Hz) showed a transient drop followed by full recovery. **(B)** Group summary (n = 7) of initial RBF responses across stimulation frequencies. All frequencies from 20 – 1000 Hz significantly reduced flow relative to the no-stimulation control condition (*p < 0.05, ***p < 0.001) except for the 15000 Hz case. Statistical comparisons were performed using Kruskal–Wallis followed by Wilcoxon post hoc tests against the control condition. **(C)** Direct comparison between 20 Hz and 15000 Hz stimulation showed significantly greater flow suppression at low-frequency stimulation (***p < 0.001).

To investigate the potential for frequency-dependent effects of electrical stimulation on renal hemodynamics, we analyzed initial response changes in renal blood flow during stimulation across a range of pulse train frequencies (from 20 Hz to 15000 Hz). Renal nerve stimulation frequencies were randomly applied each lasting 30 – 60 seconds while the blood flow response to stimulation was measured through a renal flow probe positioned over the renal vein. At a stimulation frequency of 20 Hz, a substantial reduction in renal blood flow was observed during the stimulation period (Figure 2A). In contrast, stimulation at 1000 Hz elicited a modest and transient decrease in blood flow followed by a partial recovery during stimulation.

Frequency sweep experiments showed that 20 – 200 Hz conditions produced significant reductions in renal blood flow compared to the control condition. These reductions differed in magnitude but were consistent in direction, suggesting a frequency-dependent reduction in renal blood flow with renal nerve activation (Figure 2B). In contrast, KHF (1000 – 15000 Hz) resulted in transient, smaller decreases in renal blood flow with stimulation. Statistical analysis revealed that all stimulation groups (except 15000 Hz) significantly differed from the control for the initial response (Wilcoxon test with Bonferroni correction, *** p < 0.001).

Comparing the initial blood flow responses of 20 Hz to 15000 Hz stimulation, the difference between these two conditions was highly significant (***, p < 0.0001), with 20 Hz causing a median decrease of approximately 8%, while 15000 Hz had minimal effect (−0.2%) (Figure 2C). These results supported the first hypothesis that low-frequency stimulation decreases renal blood flow and suggested that kilohertz-frequency stimulation has a distinct, non-reducing effect on renal hemodynamics in this experimental setup.

### Extended blood flow responses adapted to higher stimulation frequencies

**Figure 3.**
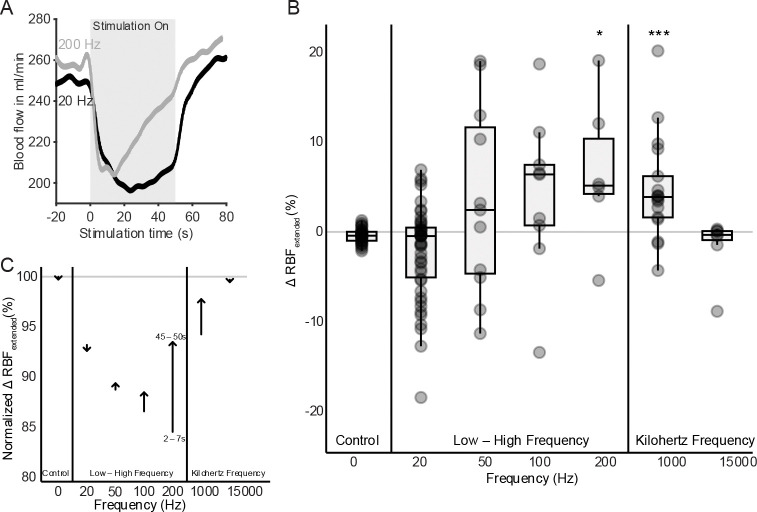
Frequency-dependent extended changes in renal blood flow (RBF) during renal nerve stimulation. **(A)** Representative RBF traces from a single subject during 20 Hz and 200 Hz stimulation. While both caused early reduction in blood flow, 200 Hz showed a slow recovery during the 50-second stimulation period. **(B)** Group summary (n = 7) of extended RBF responses across stimulation frequencies. Both 200 Hz and 1000 Hz significantly increased flow relative to the initial suppression, indicating partial recovery (* p < 0.05, *** p < 0.001). Statistical comparisons were performed using Kruskal–Wallis followed by Wilcoxon post hoc tests against the control condition. **(C)** Frequency-dependent adaptation profiles derived from the group median of normalized RBF values at initial (2 – 7 s) and extended (45 – 50 s) time points during stimulation. Arrows represent the direction and magnitude of change for each frequency band, with arrow tails marking the initial response and arrow heads representing the extended response. Mid-frequency stimulation (50–1000 Hz) showed the clearest evidence of adaptation, characterized by partial or full recovery of blood flow.

To assess the temporal dynamics of renal blood flow during stimulation, we compared the mean renal blood flow values at early (2 – 7 seconds) and later (45 – 50 seconds) time points during stimulation (Fig. 3). This measure was expressed as a percentage change and as a normalized value (relative to pre-stimulation baseline: −10 to −5 seconds).

While both 20 Hz and 200 Hz stimulation resulted in renal blood flow reductions, 20 Hz stimulation resulted in a sustained reduction, and 200 Hz showed a recovery in blood flow toward its baseline flow level (Figure 3A). High frequency stimulation (200 Hz) was associated with significant adaptation in renal blood flow (median ~ 6%; *, p < 0.05) (Figure 3B). KHF yielded a mixed effect with 1000 Hz resulting in a significant adaptation effect while 15000 Hz had less change in flow over the stimulus duration (***, p < 0.001) due to a limited change in blood flow during the initial response period.

To control inter-animal variability, normalized long-term flow responses were averaged. Stimulation frequencies between 50 – 1000 Hz showed a consistent positive change in flow between initial and late time periods, while 20 Hz and 15000 Hz showed a minimal adaptation effect as shown in Figure 3C. Arrow annotations indicate flow direction and magnitude for each frequency group, reinforcing the frequency-dependent divergence in vascular response.

### KHF induces a carryover effect on low-frequency stimulation

**Figure 4.**
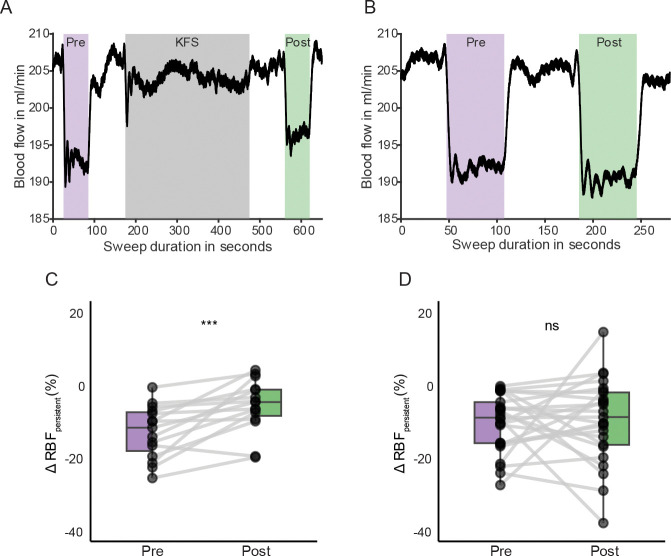
Changes in renal blood flow (RBF) to low-frequency stimulation before and after kilohertz-frequency stimulation (KHF). **(A)** Representative RBF traces from KHF trial showing low-frequency stimulation before KHF (purple), during KHF (dark gray), and after KHF (green). Flow suppression during low-frequency stimulation was attenuated after KHF delivery. **(B)** Control trial without KHF shows a consistent suppression in both low-frequency periods. **(C)** Group summary (n = 7) of normalized RBF change during low-frequency stimulation before and after KHF. Post-KHF responses showed a significantly reduced suppression compared to pre-KHF (***p < 0.001, paired Wilcoxon signed-rank test), suggesting a carryover effect. **(D)** In control trials, no significant difference was observed between the pre- and post-KHF low-frequency stimulation periods. All flow responses were averaged over the first 30 seconds and normalized to a baseline period (−10 to −5 s) for each low-frequency stimulation trial.

To determine whether KHF has carryover effects in responsiveness to renal nerve activation, we compared renal blood flow changes during repeated low-frequency (20 or 50 Hz) trials performed before and after KHF exposure. The first 30 seconds of each low-frequency stimulation period were averaged and normalized to pre-stimulation baseline (−10 to −5 seconds). The summary of renal blood flow responses comparing experiments with KHF (left panels) and without KHF (right panels) are shown in Figure 4.

Renal blood flow decreased during both low-frequency stimulation trials, but the trials immediately following KHF showed a muted decrease in renal blood flow (Figure 4A, C). Indeed, a significant difference was observed between pre- and post-KHF trials (***, p < 0.001), with post-KHF trials showing reduced suppression in flow. It is also worth noting that the observed reduction in flow occurred with low-frequency stimulation, which did not exhibit the adaptation phenomenon during prolonged stimulation as shown in Figure 3. These post-KHF effects were observed across subjects and were not observed in control experiments when KHF was not delivered (Figure 4B, D). In control experiments, no significant difference in renal blood flow was found between pre-KHF and post-LFS low-frequency stimulation trials (Figure 4D). This suggested that the observed modulation in Figure 4C was specific to the presence of KHF and not due to time or repeated stimulation. Together these findings indicate that KHF may attenuate the vasoconstrictive effect of renal nerve activation (Figure 4C).

## Discussion

This study provides new insights into the frequency-dependent modulation of renal blood flow via electrical stimulation of the renal nerves. We observed both initial and extended response changes in renal perfusion across a spectrum of stimulation frequencies. Low-frequency stimulation (e.g., 20 Hz) resulted in a robust and sustained reduction in renal perfusion, consistent with activation of efferent sympathetic fibers. In contrast, high to kilohertz-frequency stimulation (e.g., 1000 Hz) exhibited a distinct biphasic profile, characterized by an initial decrease in flow followed by partial or complete recovery. This observed neural adaptation may stem from altered nerve excitability or more network-level changes in the brain-kidney axis. Collectively, these results highlight both the critical role of renal nerve stimulation frequency on modulating renal blood flow and the potential use of renal nerve stimulation to assess target engagement for RDN therapies.

### Effects of frequency stimulation on renal blood flow

Electrical stimulation of the renal nerves causes vasoconstriction and reduces renal perfusion through activation of postganglionic sympathetic efferents(8). This leads to the release of norepinephrine, which drives renin-angiotensin-aldosterone system activation integral to the autonomic regulation of renal function, blood volume, systemic vascular resistance, and ultimately blood pressure(5,8). One consistent finding in preclinical studies is that renal sympathetic nerve stimulation reduces renal blood flow (RBF) and can lower the glomerular filtration rate (GFR), due to vasoconstriction of the afferent renal arteriole. These effects are highly frequency- and amplitude-dependent. At low physiological stimulation frequencies (~0.5–2 Hz in rats), sympathetic activation primarily triggers renin release and enhances sodium reabsorption with minimal effects on RBF or GFR(9,23). As stimulation frequency increases into a higher range (e.g. 2–6 Hz in rats), greater vasoconstriction ensues: RBF falls and renal vascular resistance rises, accompanied by further increases in renin secretion and reductions in sodium excretion. These graded responses reflect the hierarchical recruitment of sympathetic actions: low-level activity preferentially triggers beta-adrenergic mediated renin release, whereas higher levels recruit alpha-adrenergic vasoconstriction in renal arteries and arterioles, reducing perfusion and filtration(24). Our findings align with these previous studies, confirming that low-frequency stimulation (particularly at 20 – 50 Hz) consistently reduced renal blood flow, reflecting excitation of renal sympathetic fibers regulating kidney function. Cross-species studies support these findings; rabbits exhibit modest decreases in RBF and GFR with significant renin elevation and sodium retention(25), and sheep demonstrate similar acute and chronic responses(26), underscoring conserved sympathetic mechanisms across species.

Recent rodent studies have investigated low-level renal nerve stimulation to activate reflexes rather than drive efferent fibers. Salman et al. applied low-amplitude electrical pulses (0.5 mA, 0.5 ms) at 2 – 5 Hz to anesthetized spontaneously hypertensive rats. At 2.5 Hz, this protocol reduced mean arterial pressure by 25 – 35 mmHg with minimal impact on renal cortical blood flow. However, at 5 Hz, while blood pressure dropped further, renal blood flow transiently decreased, and vascular resistance increased, likely due to recruitment of efferent fibers causing vasoconstriction(27). The net effect on systemic sympathetic nerve activity and blood pressure depends on a balance between direct efferent activation (which tends to raise BP) and afferent-mediated reflexes (which can either raise or lower BP)(28). In our study, renal nerve activation, and the subsequent initial response, aligned with very minor changes in systolic blood pressure (**Supplementary Figure 1**). In both pigs and dogs, unilateral or especially bilateral renal nerve stimulation acutely raises systemic arterial pressure (pressor response) without large reflexive changes in heart rate(13,29,15,30) as shown in **Supplementary Figure 2**. Such pressor responses have even been used clinically (in catheter-based renal denervation procedures) as a diagnostic endpoint to confirm the presence of functional renal sympathetic nerves prior to ablation(12,14,29).

While renal sympathetic innervation has been extensively characterized, the presence and functional relevance of parasympathetic innervation to the kidney remains debated. DiBona and Kopp noted that while cholinergic receptors are present in the renal tissue, there was no compelling evidence at the time for cholinergic innervation in the kidney(9). However, recent anatomical and molecular studies have identified cholinergic fibers in perivascular renal nerves of humans using post-mortem immunohistochemistry(31) and in genetically modified mouse models(32), suggesting that parasympathetic renal innervation may exist. The clinical significance of these parasympathetic fibers is not fully understood – they might modulate renal blood flow or have a renoprotective role. Functionally, studies of RNS in humans and large animals have identified both pressor and depressor (BP-lowering) responses, consistent with the idea that renal nerve contains multiple fiber types(28,33,34). We observed this in our experiments, where some stimulation caused either a pressor or depressor effect (**Supplementary Figure 3**); however, these blood pressure changes were small (<4 mmHg), which is expected given the use of healthy animals and the application of unilateral stimulation. These effects nonetheless suggest the possibility of parasympathetic or indirect sympathoinhibitory effect through reflex pathway involvement—a nuance that purely sympathetic models may not capture.

### Neural adaptation and frequency-dependent responses

The prolonged delivery of renal nerve stimulation exhibited signatures of adaptation in terms of changes in renal blood flow. At higher frequencies, particularly >100 Hz range, blood flow initially decreased but then rebounded towards baseline after ~10–20 seconds. These findings underscore that sustained high-frequency renal sympathetic activity activates intrinsic feedback processes that protect against prolonged vasoconstriction despite continued sympathetic input. The results are also consistent with a previous study(35) in dogs showing that for continuous 3.3 Hz stimulation of the greater splanchnic nerve, renal blood flow initially declined (~33%) and then partially recovered over time despite continuous stimulation. In that study, sodium excretion remained markedly suppressed (<5% of control), and renin secretion remained elevated (~8-fold baseline) throughout prolonged stimulation suggesting that dynamics between renal blood flow and renal chemical release may not always parallel each other.

The phenomenon of end effector organ adaptation with prolonged delivery of stimulation is consistent with studies from both the peripheral and central nervous systems(36). This observed adaptation may involve several interacting mechanisms. One prominent candidate is the local accumulation of extracellular potassium ions which increases neuronal activation thresholds and causes temporary depolarization block. This block occurs due to the inactivation of voltage-gated sodium channels preventing further action potential generation. Additionally, synaptic mechanisms, such as neurotransmitter depletion (e.g. of norepinephrine at the neuroeffector junction) and postsynaptic receptor desensitization could also contribute significantly to the diminished renal nerve stimulation over time. Alternatively, differential recruitment and susceptibility to adaptation among various axons (small vs. large fiber types) may explain the distinct responses across low, high, and kilohertz frequencies.

### Carryover Effects of Kilohertz Frequency Stimulation

A significant finding of this study was the carry-over effect of kilohertz frequency stimulation on renal blood flow, which was assessed by comparing responses to low-frequency stimulation before and after KHF. Specifically, after KHF stimulation, subsequent low-frequency stimulation elicited attenuated vasoconstrictive responses compared to pre-KHF measurements. This observation suggests that KHF induces lasting physiological alterations, potentially through a transient axonal conduction block or modulation of synaptic channels. Previous work demonstrated that KHF can reversibly block nerve conduction in peripheral systems by disrupting action potential propagation in large-diameter axons(37,38). However, its effect on autonomic fibers innervating visceral organs – such as the renal nerves – remains underexplored.

The carryover phenomenon we observed cannot be fully explained by neurotransmitter depletion alone (i.e. a synaptic mechanism). KHF by itself did not evoke a large initial period reduction in blood flow (unlike low-frequency stimulation). If synaptic depletion were the primary factor, one might expect an initial strong vasoconstriction during KHF that then fades; instead, we observed only a minimal initial change at 1000 Hz and no change at 15000 Hz. Yet, in both cases, there was still a reduction in the renal blood flow response to low-frequency stimulation after KHF. Thus, a more likely scenario is that KHF caused a depolarization block of sympathetic fibers, silencing their activity during the stimulation period and persisting for a brief period thereafter. In this way, the weaker response may stem from fewer fibers available to be activated immediately after the KHF exposure.

If a nerve block condition were induced, however, one might expect to also observe an increase in renal blood flow or a decrease in systemic blood pressure. The lack of such response may have stemmed from weak intrinsic renal nerve activity, potentially due to the experimental preparation (e.g. anesthetized model or testing in non-hypertensive subjects). Future studies employing direct electrophysiological recordings from the renal nerve during and after KHF stimulation would be highly informative. Such data could confirm the presence of ongoing action potential firing (or lack thereof) and help dissociate axonal vs. synaptic contributions to the carryover effect. Regardless of mechanism, the ability of a KHF stimulus to transiently dampen the renal sympathetic response is of considerable interest for therapeutic neuromodulation, as it points to the possibility of reversible nerve blocks in autonomic pathways.

### Implications for Bioelectronic Control

Our findings contribute to the growing body of research on bioelectronic modulation of renal function. The ability to modulate renal nerve activity in a frequency-specific and temporally structured manner has important implications for bioelectronic control of renal and cardiovascular function. In particular, the carryover effects observed with KHF highlight the possibility of sustained and reversible neuromodulation, offering a novel strategy for peripheral nerve-targeted therapies. This is especially relevant in the context of hypertension, where reducing sympathetic outflow to the kidney can improve both blood pressure and renal function. Future studies may wish to explore the long-term effects of KHF and its potential integration into clinical interventions aimed at renal denervation and autonomic modulation.

### Limitations and Future Directions

While our study provides insight into the frequency-dependent effects of renal nerve stimulation, several limitations should be acknowledged. First, all experiments were conducted on healthy animals. This setup does not fully capture the pathophysiological features of drug-resistant hypertension or chronic kidney disease, where baseline sympathetic tone, vascular remodeling, and renal autoregulatory mechanisms may differ. As such, the translational applicability of our findings to diseased models remains to be determined. Second, all stimulations were applied unilaterally, which may underestimate the full hemodynamic impact that bilateral stimulation or systemic modulation would elicit. Furthermore, isoflurane increases renal sympathetic nerve activity(39) and may blunt autonomic reflexes (including renal vascular tone and excretory responses) to stimulation under our anesthetic regimen. Future studies should investigate these effects under conscious or minimally sedated conditions to better model physiological states. Additionally, we measured renal venous flow rather than arterial flow. In intact kidneys, venous flow generally mirrors arterial inflow because urinary output is only a fraction of total renal blood flow(40). However, stimulation- or anesthesia-induced changes in diuresis can shift venous flow independent of arterial inflow. In rats, renal nerve stimulation reduced urine glucose excretion at low frequencies but increased at kilohertz frequencies(41). Thus, some stimulation-evoked changes in our venous RBF (or lack thereof) could reflect altered urine output rather than true changes in arterial inflow.

Electrical stimulation was delivered at the junction of the descending aorta and renal artery without isolating the artery, preserving the surrounding connective tissue that contains renal sympathetic nerves. While this anatomically relevant approach enabled engagement of perivascular nerves, it also raises the possibility of current spread to vascular smooth muscle. We observed an immediate decrease in renal blood flow at stimulation onset (for frequencies > 50 Hz), followed by a gradual return toward baseline during stimulation. The rapid onset suggests a possible contribution of direct smooth muscle depolarization(42), while the subsequent neural adaptation in blood flow more closely resembles a neural mechanism. Vascular smooth muscles are specialized for sustained, tonic force (latch-bridge, Ca^2+^ handling) and therefore do not typically exhibit rapid adaptation/fatigue during maintained depolarization(43,44). In contrast, neural responses to prolonged stimulation can diminish over time due to synaptic fatigue, neurotransmitter depletion, or reduced nerve excitability(45). These dynamic changes imply that sympathetic nerves were likely engaged, but the initial response may have included non-neural components. Furthermore, without pharmacological blockade, denervation, or electrophysiological recordings, we cannot definitively attribute the observed responses to neural versus myogenic mechanisms.

To address these limitations, future studies may wish to explore the effects of frequency-specific stimulation in hypertensive and chronic kidney disease animal models, perform bilateral stimulation protocols, and assess changes through direct neural recordings. Chronic stimulation paradigms and hemodynamic outcomes (e.g., blood pressure regulation, sodium balance) will be essential for establishing the therapeutic potential of kilohertz-frequency nerve block for renal system modulation. Moreover, integration of this approach into closed-loop systems guided by physiological feedback could lay the groundwork for next-generation, patient-tailored bioelectronic therapies.

## Conclusions

This study demonstrates that renal nerve stimulation elicits frequency-dependent effects on renal blood flow, with distinct temporal and physiological profiles at low, high and kilohertz-range frequencies. Stimulation frequencies elicited distinct renal blood flow patterns. While low-frequency stimulation primarily activated vasoconstrictive processes, increasing the frequency resulted in significant neural adaptation and persistent effects after stimulation was discontinued. The latter observation that KHF can induce carry-over effects may have utility as a reversible, non-destructive form of bioelectronic medicine for the kidney that is targeted, tunable, and temporally adaptive.

Together, these findings highlight the divergent physiological effects of low- versus high-frequency stimulation on blood flow to the kidney and suggest a need to further explore the effects of kilohertz-frequency stimulation. The data supported the prediction that higher-frequency (1000 Hz) renal nerve stimulation generated an initial decrease in renal blood flow, but the lack of increase in renal blood flow over the duration of stimulation could reflect a lack of blocking effect or a blocking effect without significant endogenous signaling through the renal sympathetic nerves.

## Supplementary Material

Supplementary Files

This is a list of supplementary files associated with this preprint. Click to download.

• Supplementaryfigures.docx

## Data Availability

The datasets generated and analyzed during the current study are available on GitHub: https://github.com/NRTL-Repository/Kwaku2025_RenalNerveStim

## Abbreviations:

BP: blood pressure
GFR: glomerular filtration rate
KHF: kilohertz-frequency
LFS: low-frequency stimulation
RDN: renal denervation
RBF: renal blood flow
